# Fatal refractory chronic active Epstein-Barr virus infection with hemophagocytic lymphohistiocytosis and NK/T-cell lymphoma: a case report

**DOI:** 10.1128/asmcr.00168-25

**Published:** 2026-02-09

**Authors:** Nicholas Mielke, Marlaena Nooney, Nagendra Natarajan, Rima El-Herte

**Affiliations:** 1 Department of Medicine, Creighton University School of Medicine, Omaha, Nebraska, USA; 2 Nebraska Cancer Specialists, Omaha, Nebraska, USA; 3 Department of Internal Medicine, Division of Infectious Diseases, Creighton University School of Medicine, Omaha, Nebraska, USA; Rush University Medical Center, Chicago, Illinois, USA

**Keywords:** Epstein-Barr virus, chronic active EBV, hemophagocytic lymphohistiocytosis, NK/T-cell lymphoma, case report

## Abstract

**Background:**

Epstein-Barr virus (EBV) is most often associated with asymptomatic infection or infectious mononucleosis syndrome. A rare and fatal underestimated complication is chronic active EBV infection (CAEBV) with hemophagocytic lymphohistiocytosis (HLH) and B or NK/T-cell lymphoma.

**Case Summary:**

A 28-year-old woman presented with fevers, malaise, fatigue, abdominal pain, nausea, vomiting, and episodic skin rashes. Laboratory tests revealed pancytopenia, significantly elevated liver enzymes, and serology consistent with primary EBV infection, with very high EBV DNA in serum. All other causes of hepatitis were excluded. Liver biopsy showed EBV-positive T-cells infiltrating the liver parenchyma with erythrophagocytosis, consistent with CAEBV infection with HLH. Despite aggressive interventions, the patient ultimately succumbed to grave complications and refractory disease.

**Conclusion:**

CAEBV is a rare and fatal complication of EBV. Clinician education, early recognition, and expert consultation improve outcomes. Treatment so far consists of chemotherapy followed by hematopoietic stem cell transplantation.

## INTRODUCTION

Epstein-Barr virus (EBV) infects 90% of adults worldwide by early adolescence and has a broad spectrum of clinical manifestations (1, 2). While most EBV infections are asymptomatic or self-limited, the virus can cause infectious mononucleosis syndrome (IM, lymphoproliferative disorders, hemophagocytic lymphohistiocytosis (HLH), lymphomatoid granulomatosis, lymphocytic interstitial pneumonitis, and chronic active EBV infection (CAEBV) (2–7). Lymphomas and other infections may present with IM-like symptoms (2).

The determination of the underlying cause of IM remains important for further management (8). Diagnosis of EBV infection relies on serological and molecular testing which differentiates between acute, chronic, and past EBV infections (Table 1) (8).

**TABLE 1:** Summary of the serological EBV profiles (9)

Condition	EBV VCA IgM	VCA IgG	EA-D IgG	EBV NA-1 IgG
Primary EBV infection	Elevated	Elevated	Present	Absent
Past EBV infection	Absent	Present	Absent	Present
EBV reactivation	Elevated	Elevated	Elevated	Elevated
CAEBV	Sometimes present	Very elevated	Very elevated	Variably present/absent
Nasopharyngeal carcinoma	Absent	Elevated	Elevated	Elevated

CAEBV is a rare and frequently underrecognized syndrome. The clinical course of CAEBV varies from indolent to rapidly progressive HLH, multiorgan failure, or progression to lymphoma and death with missed or delayed diagnosis (3, 7, 10–12).

To help distinguish it from other similar conditions, four diagnostic criteria for CAEBV were proposed: persistent or recurrent IM-like symptoms for more than 3 months, detection of an increased number of EBV genomes in peripheral blood and/or affected tissues, detection of EBV-infected T or NK cells in peripheral blood and/or affected tissues, and chronic illness that cannot be explained by other known disease processes at the time of diagnosis (13, 14).

## CASE PRESENTATION

A 28-year-old woman initially presented with several weeks of persistent malaise, fatigue, intermittent fevers, chills, episodic skin rashes, generalized abdominal pain, nausea, and vomiting. Her past medical, surgical, and social history is not relevant to her current presentation.

Physical examination revealed fevers, scleral icterus, and tenderness in the right upper abdominal quadrant. Evaluation demonstrated persistent pancytopenia and significantly elevated liver enzymes. Autoimmune hepatitis workup was negative. Laboratory results are summarized in Table 2. A few days later, her symptoms and liver enzymes improved spontaneously, and she was discharged home.

**TABLE 2:** Key laboratory values and microbiology testing across the three healthcare encounters

Parameter	1st encounter	2nd encounter	3rd encounter
Laboratory values [reference range]			
WBC [4.0–12.0 k/uL]	1.3 (L)	1.7 (L)	1.2 (L)
Absolute neutrophil count k/uL (%)	700 (54%)	900 (52%)	700 (58%)
Absolute leukocyte count k/uL (%)	400 (33%)	600 (33%)	400 (31%)
Hemoglobin [12.0–16.0 gm/dL]	12.1	11.5 (L)	11.5 (L)
Platelets [140–440 k/uL]	97 (L)	132 (L)	162
Alkaline phosphatase [33–138 u/L]	293 (H)	340 (H)	367 (H)
AST [10–40 u/L]	1,411 (H)	1,352 (H)	2,916 (H)
ALT [12–78 u/L]	1,865 (H)	1,381 (H)	2,302 (H)
Bilirubin, direct [0.0–0.3 mg/dL]	3.0 (H)		
Bilirubin, total [0.0–1.5 mg/dL]	3.7 (H)	3.5 (H)	3.3 (H)
Microbiology testing			
Cytomegalovirus (CMV)			
IgG	Detectable		
IgM	Undetectable		
HIV 5th generation screen and NAAT	Negative & undetectable	Negative & undetectable	Negative & undetectable
Hepatitis A/B/C serological screen and NAAT	Negative	Negative	Negative
Epstein-Barr virus (EBV)			
Viral capsid antigen IgM [0.0–43.9]		12.0 U/mL	Undetectable
Viral capsid antigen IgG [0.0–21.0]		>750.0 U/mL	Detectable
Nuclear antigen IgG [0.0–21.9]		118.0 U/mL	Undetectable
Early D antigen IgG [0.0–10.9]		>150.0 U/mL	Detectable
DNA quantitative in plasma		11,800 IU/mL	50,200 IU/mL

One month later, she was readmitted after outpatient labs demonstrated persistent pancytopenia and transaminitis. A liver biopsy demonstrated EBV-related hepatitis. Then, additional EBV serology showed detectable EBV viral capsid antigen (EBV VCA) IgG, EBV nuclear antigen (EBV NA) IgG, and EBV early antigen (EBV EA) antibodies. EBV VCA IgM was detected below the limit of the assay. EBV DNA in serum was 11,800 IU/mL. Symptoms improved spontaneously, and she was discharged.

A few weeks later, she was readmitted for persistent fevers, worsening transaminitis, and persistent pancytopenia. Repeat serology showed detectable VCA IgG and Early D antigen IgG with loss of EBV VCA IgM and EBV NA IgG. EBV DNA increased to 50,200 IU/mL. Following improvement, she was discharged with close follow up. A bone marrow biopsy was done and showed a minor T-cell clone without overt HLH.

Review of the liver biopsy at a reference lab showed core biopsies of liver parenchyma with sinusoidal dilation that contained small sized lymphoid cells lacking overt cytologic atypia better appreciated by immunohistochemistry. There was mild lobular inflammation without overt parenchymal damage. There was evidence of erythrophagocytosis seen in focal sinusoidal Kupffer cells. Submitted immunohistochemical stains and special stains were reviewed (Epstein-Barr encoded RNA [EBER], reticulin, HSV1, trichrome, iron, PAS, CMV). Additional stains performed (EBER/ CD79 and EBER/ CD3 double stains, EBER by ISH, CD20, CD3, CD4/8 double stain, and CD163) showed CD3 highlights most of the lymphoid cells which were predominantly positive for CD4, with rare cells positive for CD8 immunohistochemistry. EBER by ISH was positive in many of the lymphoid cells, and by double stains, it showed EBER positivity in the CD3-positive T-cells. CD20 highlights scattered B cells. Reticulin showed relatively preserved hepatic parenchyma. Immunohistochemical stains for Cytomegalovirus and Herpes simplex virus-1 were negative. The remaining stains were negative or non-contributory. Overall findings of the liver showed EBV-related hepatitis with evidence of erythrophagocytosis and EBV positivity in the T lymphoid cells. With these findings, the patient was diagnosed with HLH and CAEBV.

**Fig 1:**
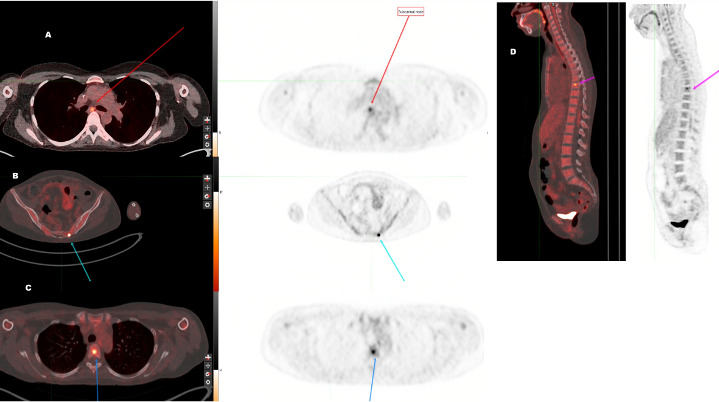
PETCT scans. Picture A shows the subcarinal lymphnode at the red arrow, picture B shows the sacral lesion at the green arrow, and pictures C and D show the vertebral lesions at the blue and purple arrows.

HLH-94 protocol (etoposide, dexamethasone) was started. She did not achieve remission. She was referred to a tertiary care center for allogeneic hematopoietic stem cell transplantation (AlloHSCT). Evaluation prior to AlloHSCT showed persistent pancytopenia, transaminitis, triglycerides 590 mg/dL (<150 mg/dL), ferritin 723 ng/mL (<120 ng/mL for female), fibrinogen 133 mg/dL (200–400 mg/dL), EBV 69,012 IU/mL, CXCL9 27,650 pg/mL (<647 pg/mL), TNF- α 33 pg/mL (<7.2 pg/mL), IL-10 84.7 pg/mL (<7 pg/mL), IL-18 2,965 pg/mL (<468 pg/mL), IL-2R 1,157 pg/mL (175.3–858.2 pg/mL). Positron Emission Tomography showed hypermetabolic subcarinal and hilar nodes (standardized uptake value [SUV] of 5.1 and 14.7, respectively), hypermetabolic ground glass opacities in the left lower lung, and multiple hypermetabolic bony foci with SUVs up to 9.5 (left sacrum lesion), and splenomegaly (Fig. 1). The brain showed a focus of increased FDG activity on the surface. Cerebrospinal fluid cytology and flow cytometry were negative for malignant cells.

High-dose methylprednisolone was initiated and rapidly tapered, followed by ruxolitinib, resulting in transient improvement (EBV DNA briefly reduced to 1,020 IU/mL and liver enzymes normalized). She underwent AlloHSCT from a haploidentical EBV seropositive donor. Her post-transplant course was complicated by BK virus cystitis, subarachnoid hemorrhage, and NK cell expansion. Despite subsequent treatment with chemotherapy (dexamethasone, methotrexate, ifosfamide, L-asparaginase, etoposide [mSMILE]) and two donor leukocyte infusions, HLH remained refractory, and NK cells continued to expand. She remained pancytopenic; her liver enzymes continued to rise. She developed lung nodules. A second HSCT was planned but not performed due to disease progression. Ultimately, the patient died due to refractory HLH, uncontrolled NK cell expansion, and secondary opportunistic infections within 1 year since symptoms onset.

## DISCUSSION

### Overview of CAEBV

CAEBV is a rare, life-threatening disorder characterized by persistent or recurrent IM-like symptoms, elevated EBV DNA in serum, and clonal proliferation of EBV-infected B, T, or NK cells. CAEBV often progresses to HLH and lymphoma (4, 12, 14). This case illustrates the aggressive clinical course of CAEBV complicated by HLH and NK cell expansion and highlights the diagnostic and therapeutic challenges in managing this disease.

### Diagnosis and serologic patterns

Diagnosis of EBV infection is established by history, physical exam, and serological testing (8). In the USA, EBV-specific antibody panels typically test for antibodies to VCA, EA, and EBV NA. The heterophile antibody test has limited sensitivity and specificity; false-negative results occur in ~10% of cases (8). More definitive evaluation relies on EBV-specific antibody testing (Table 1) (9).

CAEBV follows an inadequately controlled acute primary infection or reactivation linked to immune dysregulation or genetic susceptibility (9). In our patient, markedly elevated VCA IgG and EA IgG with absent EBV NA IgG, high plasma EBV DNA, and an IM-like presentation (fever, hepatitis, cytopenias) is most consistent with primary EBV infection progressing to CAEBV. The absence of VCA IgM does not rule out acute infection due to its transient nature and possible assay or timing limitations. Notably, the patient initially had EBV NA IgG that later became undetectable, suggesting loss of immune control.

### Epidemiology, clinical features, and pathogenesis

CAEBV is a heterogeneous disorder with variation in cell lineage, age of onset, and clinical presentation across different regions (12). Table 3 compares the Asian and the US differences of CAEBV. Clinically, CAEBV can involve any organ (15).

**TABLE 3:** Clinical and epidemiologic differences in CAEBV by geographic region (12)

Feature	Asia (Japan, East Asia, some Latin America)	United States/Western countries
Predominant infected cell type	Predominantly T-cells or NK cells; B-cell disease is uncommon	Mixed; B-cell disease common, but T- or NK-cell CAEBV also reported
Geographic distribution	Markedly enriched in East Asia (Japan, China, Korea) and also reported in Latin America; patterns suggest possible genetic predisposition	Rare overall; smaller cohorts and registries with mixed B- and T/NK-cell phenotypes
Age at onset	Often pediatric or young adult; large Japanese series show median onset in adolescence/young adulthood, many <20 years	Broader age range with substantial adult onset; B-cell-predominant cases often present later
Presenting symptoms	Persistent/recurrent fever, lymphadenopathy, splenomegaly, hepatitis, cytopenias; often progresses to HLH or EBV-positive lymphoma	Fever, lymphadenopathy, splenomegaly, hepatitis, cytopenias; may progress to HLH or EBV-positive lymphoma
Curative therapy	Allogeneic hematopoietic stem cell transplantation is the only consistently curative approach	Same as Asia

Progression to HLH is driven by immune dysregulation and cytokine storm from EBV-infected lymphocytes that are clonal and harbor large genomic deletions that are detectable in blood but not saliva and persist as long as the pathological clone remains. Following effective therapy, these deletions disappear, even in the setting of viral reactivation, suggesting that monitoring for loss of such deletions may serve as a biomarker for treatment success (16).

Kaposi sarcoma herpes virus was the cause of HLH in patients with compound heterozygous mutations in the perforin gene and absent NK cell function and under steroid therapy for hemolytic anemia (17, 18).

### Treatment strategies

Management of CAEBV with HLH and lymphoma is challenging. Allogeneic HSCT is the only curative option (60% survival) which is done after HLH is controlled (5, 11, 13, 14, 19).

Bollard et al. (11) propose a structured approach for management of T-cell CAEBV: (i) confirm EBV-positive T-cell disease, (ii) assess for HLH and treat if present, and (iii) consider bridging regimens (high-dose corticosteroids, bortezomib plus ganciclovir, a histone deacetylase inhibitor plus ganciclovir, or romidepsin plus ganciclovir) in symptomatic patients without HLH to control inflammation and viral load before HSCT. If lymphoma is present, chemotherapy regimens such as CHOP (cyclophosphamide, doxorubicin, vincristine, and prednisolone), DA-EPOCH (dose-adjusted etoposide, doxorubicin, and cyclophosphamide with vincristine, prednisone), or SMILE (dexamethasone, methotrexate, ifosfamide, l-asparaginase, and etoposide) is indicated. Asymptomatic patients should be monitored closely while a suitable donor is identified. Because of the high risk of disease progression, HSCT should proceed promptly once disease control is achieved. The overarching goal of all pre-HSCT therapy is to maximize viral and disease control while avoiding irreversible organ damage that could preclude transplant. After HSCT, donor-derived EBV-specific T-cells may be considered to prevent or treat relapses.

Available antiviral will not change the course of the disease because of absent activity during the virus latent phase in the viral reservoir.

### Conclusion

CAEBV complicated by HLH with NK-cell expansion has a high mortality rate despite multimodal therapy. Optimal outcomes depend on early recognition, prompt referral to experienced centers, pre-transplant stabilization, and timely HSCT. Continued research into targeted and immunotherapeutic strategies is essential to improve survival in this aggressive disease.
